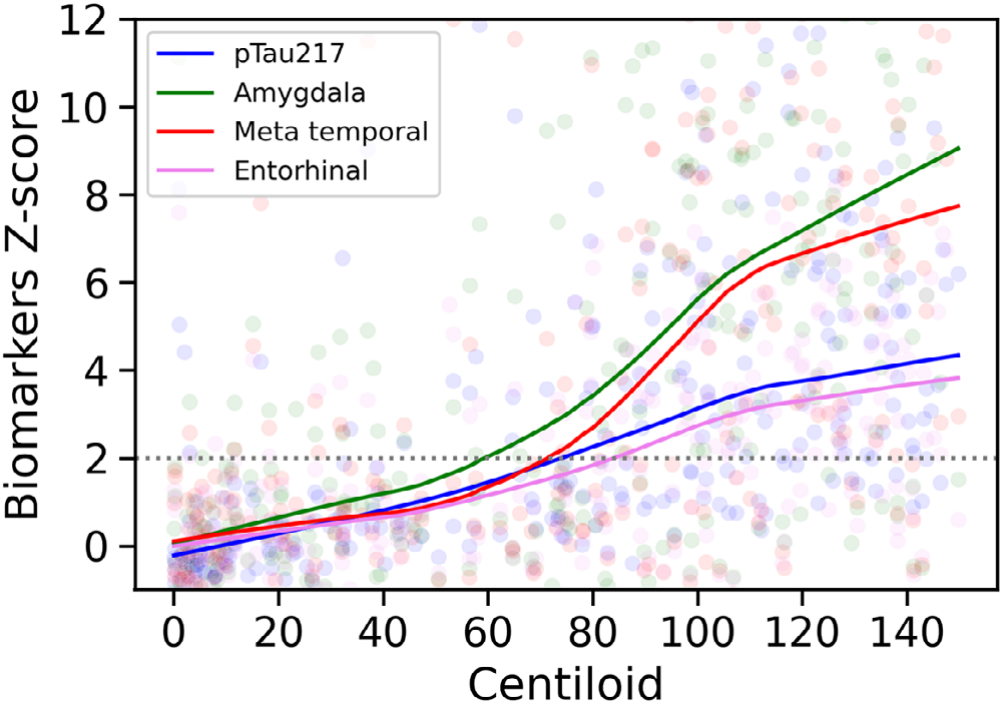# Plasma phospho‐tau 217 rises with and not before early tau aggregation measured by MK6240 PET

**DOI:** 10.1002/alz.091230

**Published:** 2025-01-09

**Authors:** Azadeh Feizpour, Vincent Dore, James D. Doecke, Colin L Masters, Christopher C. Rowe

**Affiliations:** ^1^ Department of Molecular Imaging & Therapy, Austin Health, Heidelberg, VIC Australia; ^2^ The Florey Institute of Neuroscience and Mental Health, Melbourne, VIC Australia; ^3^ Australian E‐Health Research Centre, CSIRO, Melbourne, VIC Australia; ^4^ The Australian e‐Health Research Centre, CSIRO, Brisbane, QLD Australia

## Abstract

**Background:**

Rise in plasma phospho‐tau (pTau) is hypothesized to reflect a physiological response to brain Aβ plaques, preceding the formation of neurofibrillary tangles (NFT). An alternate explanation is poor sensitivity of tau PET for detection of early NFT formation. The tau tracer MK6240 has very low background “off‐target” binding and may better detect early tau aggregation. Choice of tau PET regions for analysis may also influence early detection of tau.

**Method:**

Participants included 105 cognitively unimpaired (CU) and 178 cognitively impaired (CI), with plasma pTau217 Lumipulse® assay, ^18^F‐NAV4694 Aβ PET and ^18^F‐MK6240 tau PET data. The entorhinal, amygdala and meta temporal (MT) ROI ^18^F‐MK6240 SUVR and pTau217 levels were modelled as a function of Centiloid (CL).

**Result:**

Rise in plasma pTau217 slightly lagged the rise in amygdala MK6240 binding and paralleled the rise in the meta temporal ROI (Figure 1), consistent with our previous report using Janssen p217+Tau measured by Simoa®^1^. Our findings also suggest that amygdala and meta temporal ROI were more robust for early detection of aggregated tau than the entorhinal cortex possibly related to PART and small ROI volume producing wide variance in the entorhinal normal range in Ab‐ CU.

**Conclusion:**

This data suggests that the amygdala is a robust region for detecting early tau aggregation by MK6240 PET and that a rise in plasma pTau217 is associated with early formation of NFT rather than a direct physiological response to brain Aβ plaques prior to NFT formation.

Figure 1 legend: Modeling of ^18^F‐MK6240 quantification and pTau217 as a function of Centiloid. Biomarker values Z‐scored using the results from Aβ‐ cognitively unimpaired to define the normal range for p217+tau level and each tau PET ROI SUVR. The horizontal line is +2 standard deviations (SD).

^1^Doré V, Doecke JD, …, Rowe CC. Plasma p217+tau versus NAV4694 amyloid and MK6240 tau PET across the Alzheimer's continuum. Alzheimers Dement (Amst). 2022 Apr 5;14(1):e12307. doi: 10.1002/dad2.12307. PMID: 35415202; PMCID: PMC8984092.